# Tomato Leaf Disease Recognition on Leaf Images Based on Fine-Tuned Residual Neural Networks

**DOI:** 10.3390/plants11212935

**Published:** 2022-10-31

**Authors:** Paul Shekonya Kanda, Kewen Xia, Anastasiia Kyslytysna, Eunice Oluwabunmi Owoola

**Affiliations:** School of Electronics and Information Engineering, Hebei University of Technology, Tianjin 300401, China

**Keywords:** deep learning, plant leaf images, disease recognition, tomato leaf

## Abstract

Humans depend heavily on agriculture, which is the main source of prosperity. The various plant diseases that farmers must contend with have constituted a lot of challenges in crop production. The main issues that should be taken into account for maximizing productivity are the recognition and prevention of plant diseases. Early diagnosis of plant disease is essential for maximizing the level of agricultural yield as well as saving costs and reducing crop loss. In addition, the computerization of the whole process makes it simple for implementation. In this paper, an intelligent method based on deep learning is presented to recognize nine common tomato diseases. To this end, a residual neural network algorithm is presented to recognize tomato diseases. This research is carried out on four levels of diversity including depth size, discriminative learning rates, training and validation data split ratios, and batch sizes. For the experimental analysis, five network depths are used to measure the accuracy of the network. Based on the experimental results, the proposed method achieved the highest F1 score of 99.5%, which outperformed most previous competing methods in tomato leaf disease recognition. Further testing of our method on the Flavia leaf image dataset resulted in a 99.23% F1 score. However, the method had a drawback that some of the false predictions were of tomato early light and tomato late blight, which are two classes of fine-grained distinction.

## 1. Introduction

We rely on edible plants in the same way that we rely on oxygen. There is no food without crops, and there is no life without food. It is no coincidence that the invention of agriculture coincided with the rise of human civilization [1]. The tomato (*Solanum lycopersicum* L.), family Solanaceae, originated in the Andean region of South America has, according to the Food and Agriculture Organization Statistics (FAOSTAT), in the past fifty years become one of the most important and extensively grown horticultural crops in the Mediterranean region and throughout the world. Currently, it is the world’s second most cultivated vegetable crop after the potato, with approximately 181 million tonnes from 5 Mha [2]. With 0.2 Mha, it is the highest-yielding vegetable in Southern Europe, and the major producers in the Mediterranean basin are Turkey, Egypt, Italy, Spain, and Morocco [2,3].

The tomato is susceptible to a variety of plant diseases caused by pathogens such as fungal, bacterial, phytoplasma, virus, and viroid pathogens due to its genetic properties as shown in Table 1. Not only is its genetic inheritance critical to managing the numerous tomato pathogens, but so are current climate changes, recently revised phytopathological control measures, and seed industry globalization [4]. One of the common diseases affecting tomato yield, the Septoria leaf spot, is caused by a fungal pathogen. Septoria has emerged as a major emerging pathogen as a result of climatic change and widespread variability. The pathogen’s disease severity ranges between 35 and 65 percent in both cultivated and non-cultivated crops, posing a serious threat in the future [5]. The pathogen’s complex adaptability combined with its cosmopolitan nature makes it more vigorous by targeting new susceptible hosts and coupling this with increased viability within its infection cycle [5]. Table 1 lists some tomato plant pathogens present in the Mediterranean basin [4].

The cultivated tomato has a low genetic diversity due to its intensive selection and severe genetic bottlenecks that arose during evolution and domestication [10,11,12]. For these reasons, the tomato is more prone to a high disease incidence, and during the cultivation and post-harvest period, it can be affected by more than 200 diseases caused by different pathogens throughout the world [13,14]. In this paper, we propose a method for effective recognition of diseases affecting the tomato, that are mainly reflected on the leaves. We propose a residual neural network algorithm for this, which is the state-of-the-art, most recent deep learning image recognition algorithm. The efficient recognition of such can inform the farmers of the presence of such diseases on their crops and allow them to carry out control measures currently authorized in the EU that allow growers to achieve a successful and eco-sustainable disease management of this vegetable crop, fundamental for the Mediterranean diet.

One of the most important research areas in precision agriculture is disease identification using images of plant leaves [15]. Artificial intelligence, image processing, and graphical processing unit advancements have the potential to broaden and improve the practice of precise plant protection and growth. Most plant diseases produce a variety of visible symptoms; thus, learning models should be able to adequately observe and identify the distinctive symptoms of any disease [16].

Tomato leaf disease identification falls under the purview of computational agriculture [17,18]. Traditional methods for identifying tomato leaf disease at an early stage frequently use global features (such as color, texture, and shape) to describe the characteristics of disease spots in crop leaf disease images. The methods used served to separate the diseased and normal parts of the tomato leaf, and the area ratio of the two parts was used as the criterion for identification [19].

Recent advancements in deep learning provide obvious benefits in feature extraction and recognition, such as the convolutional neural network (CNN), which automatically trains the network to extract data features by introducing local connections and weight sharing in the training process. Additionally, significant progress has been made in the identification of diseases in plants such as apples, bananas, cucumbers, and tomatoes [20,21].

However, the recognition effect varies due to the structural differences of the various recognition models that make up the state-of-the-art model. Additionally, there exists the challenge of choosing suitable hyperparameters for training networks on these leaf datasets while leveraging the richly learned weights that some state-of-the-art models have gotten while learning from very large datasets. Additionally, different layers need to learn at different learning rates, for an optimal learning experience.

As a result, the primary aim of this research is to achieve high recognition accuracy for tomato leaf diseases; to achieve this, a residual neural network will be utilized, and the following will be studied and modified for the proposed network:

1. A double form of data augmentation, using image transformations and the implementation of CutMix as a secondary form of data augmentation for model generalization.
2. The effect of the train/test data split size ratio of our dataset on the network model for disease recognition on tomato leaf images. Train/test data split ratios of sizes 40/60, 50/50, 60/40, 70/30, and 80/20 were adopted and studied.
3. The effect of varying batch sizes in training our network to correctly recognize tomato leaf diseases. According to the capacity of the GPU available, batch sizes of 40, 50, 60, 70, 80, 90, and 100 were adopted.
4. The role of network depth in the effective recognition of tomato leaf disease. Residual networks with varying depths of 18, 34, 50, 101, and 152 layers were studied.
5. The effect of tuning the learning rate while training the network and identification of a threshold to obtain suitable learning rates to effectively train the network to recognize tomato leaf diseases. The implementation of a discriminative learning rate for efficient training of residual models.

## 2. Related Work

Durmus et al. [22] used AlexNet [23] and SqueezeNet [24] models to classify and recognize 10 different types of tomato diseases in the PlantVillage dataset. The experiment discovered that while AlexNet’s classification accuracy is slightly higher than SqueezeNet’s, the size of the model and the time required are doubled.

Aravind et al. [25] used AlexNet and VGG16 [26] in conjunction with transfer learning to identify seven types of tomato diseases; the experiment revealed that the accuracies were 97.29 and 97.49 percent, respectively. Although transfer learning can accelerate model convergence and improve recognition performance, it is constrained by the original network structure.

Karthik et al. [27] proposed an attention-based deep residual network for detecting the type of tomato leaf infection. The PlantVillage dataset was used in the experiment, with 95,999 images used as training models and 24,001 images used for validation. The diseases included in the dataset were the tomato early blight, late blight, and leaf mold. The experimental results demonstrated that the proposed attention-based residual network can use CNN learning features at different processing levels and achieve 98 percent overall accuracy on the validation set in five-fold cross-validation.

Anand et al. [28] proposed an image processing and machine learning-based technique for diagnosing brinjal leaf disease. They used a K-means clustering technique to segment brinjal leaf diseases with some remarkable performance. In 2018, Zhang et al. [29] proposed a K-means clustering and PHOG algorithms-based fusion of super-pixel clustering-based leaf segmentation. Their technique performed admirably in the segmentation and recognition of plant leaf diseases. By extracting features based on color and texture and feeding them to a multiclass SVM classifier, Rani et al. [30] also proposed a K-means clustering-based leaf disease and classification technique. On average, they recorded a classification accuracy of 95%. In the same year, Kumari et al. [31] proposed an image processing-based leaf spot recognition system, with the four stages of image acquisition, image segmentation, feature extraction, and classification. To compute the disease features, they also utilized the K-means algorithm. They achieved an accuracy of 90% and 80% for bacterial leaf spot and cotton leaf disease target spot, respectively. Liu et al. [32] proposed a leaf disease identification model based on generative adversarial networks. This model employed DenseNet and instance normalization to recognize actual and false disease images, as well as the feature extraction capability on grape leaf lesions. Finally, the approach applied a deep regret gradient penalty to stabilize the training process. The findings revealed that the GAN-based data augmentation strategy may effectively overcome the overfitting problem in disease identification while simultaneously improving accuracy. A leaf disease detection approach based on the AlexNet architecture was proposed by Lv et al. [33] in 2020. First, they created a maize leaf feature enhancement framework, which improved the capability of feature extraction combined with dilated convolution and multiscale convolution in a complex environment. After that, a DMS-Robust AlexNet architecture network was created, which improved the capability of feature extraction combined with dilated convolution and multiscale convolution in a complex environment. The disease features on tomato leaves, such as spot blight, late blight, and yellow leaf curl disease, were extracted using a deep learning method by Jiang et al. [34]. After continuous iterative learning, the proposed technique correctly predicted the disease category for each disease, with accuracy increases of 0.6 percent and 2.3 percent in the training and test sets, respectively. Waheed et al. [35] proposed an optimized DenseNet-based maize leaf recognition model with few parameters to boost job efficiency. The results of the experiments demonstrated that this technology is effective at detecting corn leaf disease. Huang et al. [36] proposed an end-to-end plant disease diagnostic model-based deep neural network, which can reliably classify plant types and plant diseases. Their model consists of two components: the leaf segmentation part that separates the leaves in the original image from the background; and the plant disease classifier, which is based on a two-headed network that classifies plant diseases using features extracted by multiple common pre-trained models. Experimental results show that this method can achieve a plant classification accuracy of 0.9807 and a disease recognition accuracy of 0.8745. In 2020, [37] proposed a combination of ABCK-BWTR and B-ARNet models for the identification of tomato leaf disease, consisting of a channel attention module in a ResNet50 [38], using the dual channel filter to extract the primary leaf features.

Sethy et al. [39] used different deep learning models for extracting rich features and applied an SVM classifier to classify them. They achieved their highest performance accuracy with a combination of a ResNet50 model with an SVM classifier. Oyewola et al. [40] in their work proposed using plain CNNs(PCNN) and deep residual network (DRNN) in identifying five different cassava plant diseases; their results showed that PCNN was outperformed by DRNN by a margin of +9.25%. Zeng et al. [41], on the other hand, proposed a self-attention convolutional neural network (SACNN) to identify several crop diseases. To examine the robustness of their model, the authors introduced noise at different levels in the test images. Diseases prone to affect rice and maize leaves were identified by Chen et al. [42] using an INC-VGGN method. They replaced the last convolutional layer of a VGG19 model with two inception layers and a global average pooling layer. Maize, apple, and grape crop diseases were identified by Yang et al. [43] using a shallow CNN (SCNN) embedded with SVM and RF classifiers. A transfer-learning approach was adopted by Ramacharan et al. [44] to identify three diseases and two pest-damage types that plague cassava plants. The authors further extended their work by implementing a smartphone-based CNN model for the identification of cassava plant diseases and recorded an 80.6% accuracy [45]. Adedoja et al. [46] proposed a deep CNN architecture based on NASNet to identify diseases on some plant leaves with an accuracy of 93.82%. However, there is still a need for improvement in the accuracy of plant disease recognition.

In 2022, an attention-based method was proposed by Devi et al. [47] where they used the Salp Swarm algorithm in the classification of tomato leaf diseases. Their method achieved an accuracy of 97.56% in predicting five types of tomato leaf diseases from leaf images taken from the plant village dataset. Apart from the computational complexity of the method, it is also limited in performance score. A method that utilized a lightweight attention-based CNN [48] to classify tomato leaf diseases achieved a model accuracy of 99.34% but with a slightly higher time complexity than conventional methods. Also in 2022, Zhao et al. [49] developed a method that utilized a spatial attention mechanism with CNN for real-time leaf disease detection. However, this method achieved a 95.20% accuracy and did not generalize well. With the aim of improving performance and generalizability, our method was researched. We also tested the proposed method on another plant leaf benchmark dataset that is different from our target dataset.

## 3. Evaluation Metrics, Results, and Discussion

This section presents the metrics used in evaluating the results of this research, the detailed results, and relevant discussion.

### 3.1. Evaluation Metrics

The accuracy, precision, recall, and f1-score of the proposed method were all evaluated. The proposed plant recognition system’s accuracy has been calculated using the following expression, which incorporates numerical details such as true positive (TP) (the number of correctly identified leaf images), false positive (FP) (the number of incorrectly detected leaves), true negative (TN) (the number of correctly detected leaf images), and false negative (FN) (it is a parameter for representation of the number of leaf images that are correctly recognized).

- Accuracy: Accuracy is the number of right predictions that are made by the model with respect to the total number of predictions that were made. It is mathematically represented by Equation (1).

(1) $$\begin{array}{c} \mathrm{Accuracy}=\frac{\mathrm{TP}+\mathrm{TN}}{\mathrm{TP}+\mathrm{TN}+\mathrm{FP}+\mathrm{FN}} \end{array}\;$$

- Precision: Precision is defined as the number of true positive results (TP) divided by the number of positive results (TP + FP) that are predicted by the model. The range of the precision is between 0 and 1 and is calculated using Equation (2). It is used to find the proportion of positive identifications that is true.
- Recall: The recall is the number of true positives (TP) divided by the number of all relevant sample data (TP + FN). Equation (3) represents the mode of calculation of the recall. It is used to determine the proportion of actual positives that were correctly identified. These concepts are represented mathematically by Equations (2) and (3), respectively:

(2) $$\begin{array}{c} \mathrm{Precision}\;=\frac{\mathrm{TP}}{\mathrm{TP}+\mathrm{FP}} \end{array}\;$$

(3) $$\begin{array}{c} \mathrm{Recall}\;=\frac{\mathrm{TP}}{\mathrm{TP}+\mathrm{FN}} \end{array}\;$$

- F1 Score: Being one of the widely used metrics for the performance evaluation of machine learning algorithms, the F1 score is the harmonic mean of precision and recall. The range of the F1 score is between 0 and 1, and it is calculated as shown by Equation (4). It reflects the number of instances that are correctly classified by the learning model.

(4) $$\begin{array}{c} F1=\frac{2\times P\times R}{P+R} \end{array}\;$$

### 3.2. Results and Discussion

#### 3.2.1. Results on Varied Network Depth

**Figure 1:**
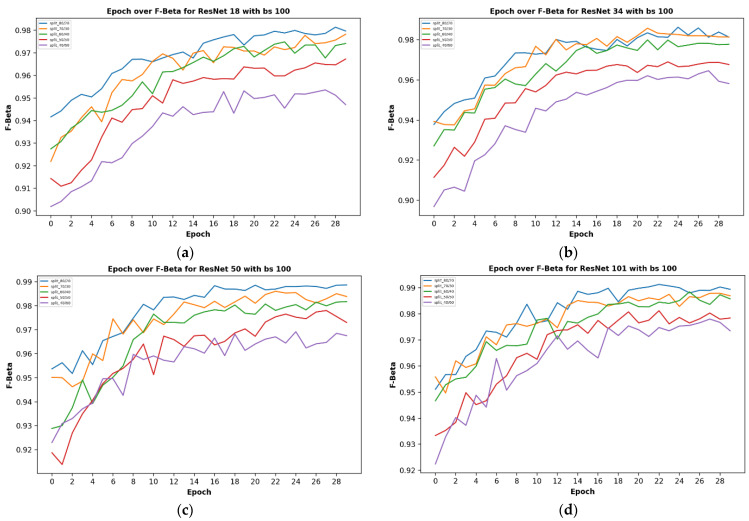

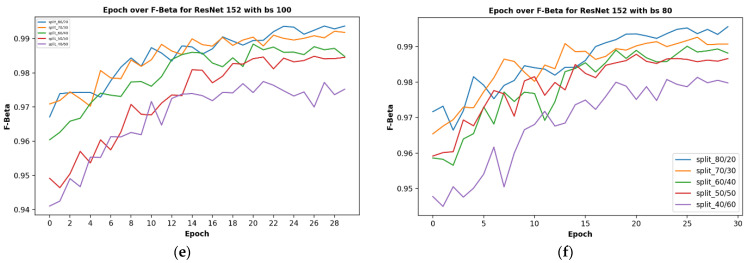
Showing the results on different train/test split ratios on various models used in our research: (**a**) showing epoch over F1 score for ResNet-18; (**b**) showing epoch over F1 score for ResNet-34; (**c**) showing epoch over F1 score for ResNet-50; (**d**) showing epoch over F1 score for ResNet-101; (**e**) showing epoch over F1 score for ResNet-152; (**f**) showing epoch over F1 score for ResNet-152 on a batch size of 80, this is where the best F1 score of our research is located.

**Figure 2:**
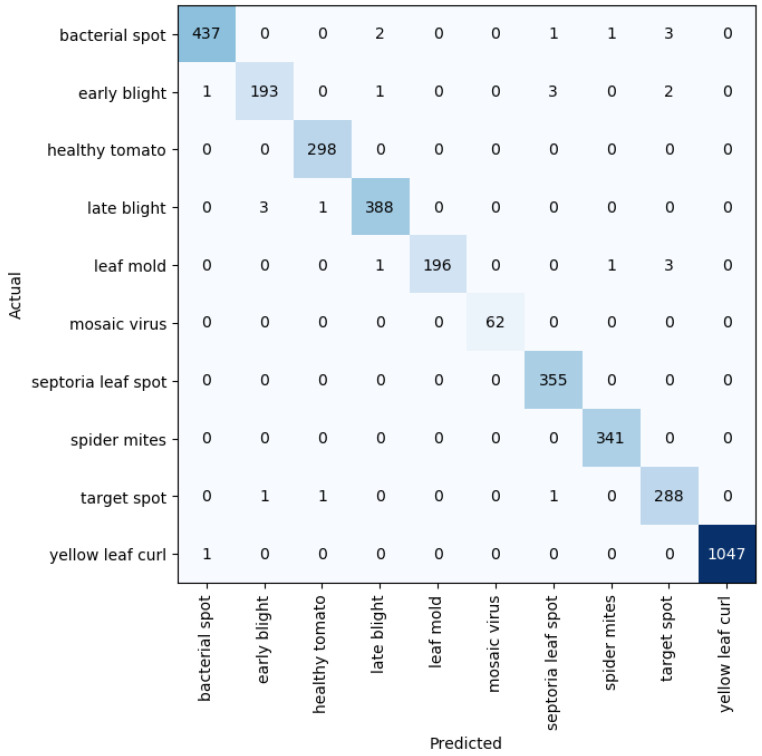
Confusion matrix for network depth = 34.

**Figure 3:**
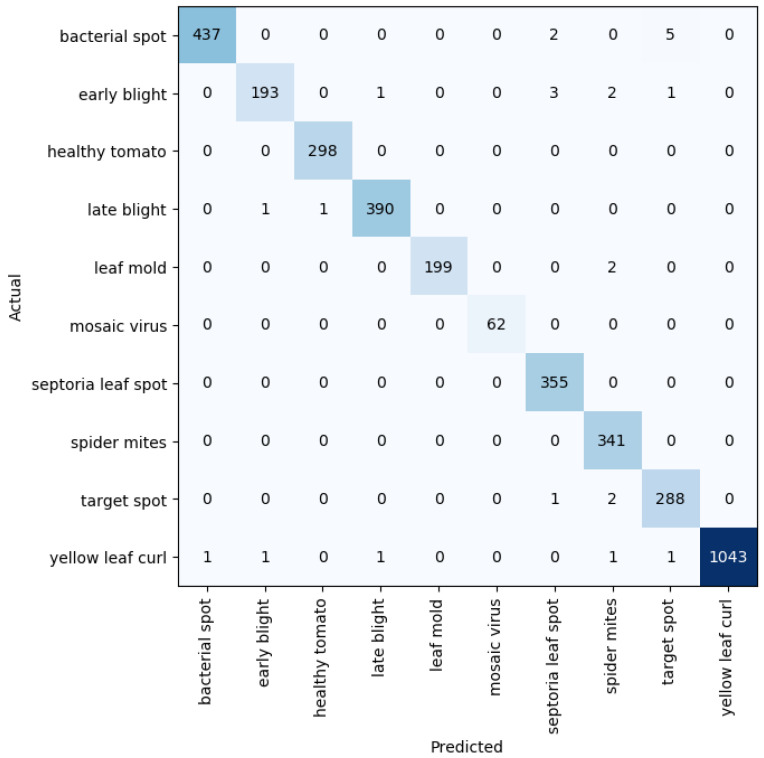
Confusion matrix for network depth = 50.

**Figure 4:**
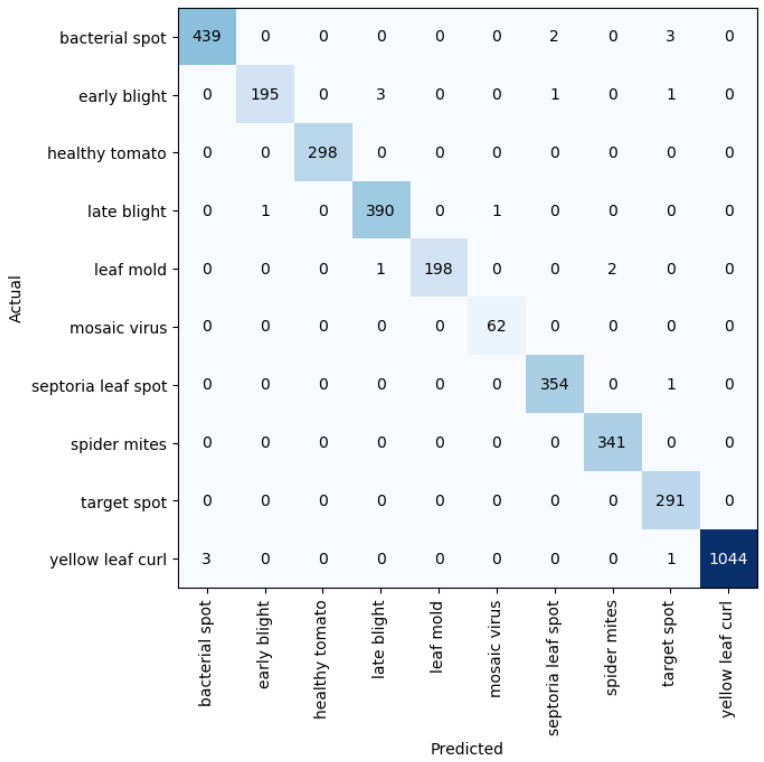
Confusion matrix for network depth = 101.

**Figure 5:**
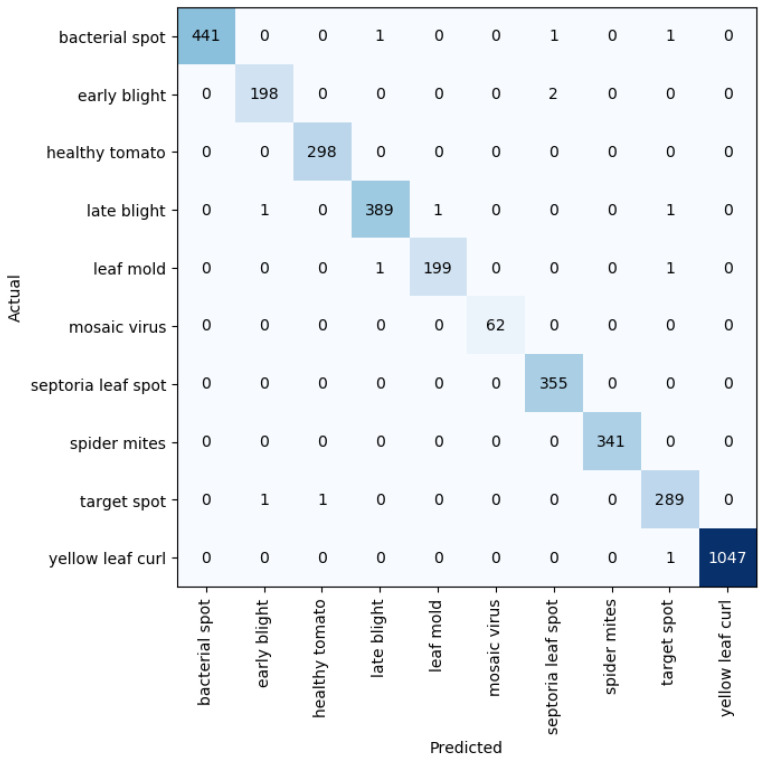
Confusion matrix for network depth = 152.

Given five different network depths adopted in this research, 18, 34, 50, 101, and 152 layers, respectively, this section reports the results and discusses the findings on the relationship between the network depth and the performance of the proposed network. The results of the F1 score based on the different depths of the proposed residual neural network are displayed in Figure 1, while Figure 2, Figure 3, Figure 4 and Figure 5 show the performance via confusion matrices of the various network depths.

Figure 1 displays the plot of epoch over F1 score for a network of the five varying depths (18, 34, 50, 101, and 152) on the maximum batch size used in this research, having a value of 100. The results show that a train-validation split ratio of 80/20 recorded the highest performance in the F1 score. After 29 epochs, the result shows how the performance score reached a peak of 98% and a minimum of 94% based on different train-validation data split ratios as indicated on the plot image.

It was observed that the network depth affected the network performance, though not at a very high value. However, the network depth of 152, being the highest depth used in this research, had the highest performance score of 99.51% as shown in Figure 1e above.

#### 3.2.2. Results on Varied Train-Validation Data Split Ratios

Different train-validation data split ratios were tested on this proposed network, ranging from 40/60, 50/50, 60/40/ 70/30, and 80/10 for training and validation data, respectively. This section displays and discusses the results obtained.

Table 2 shows the relationship between batch size and performance on the network on different train-validation data split ratios.

**Figure 6:**
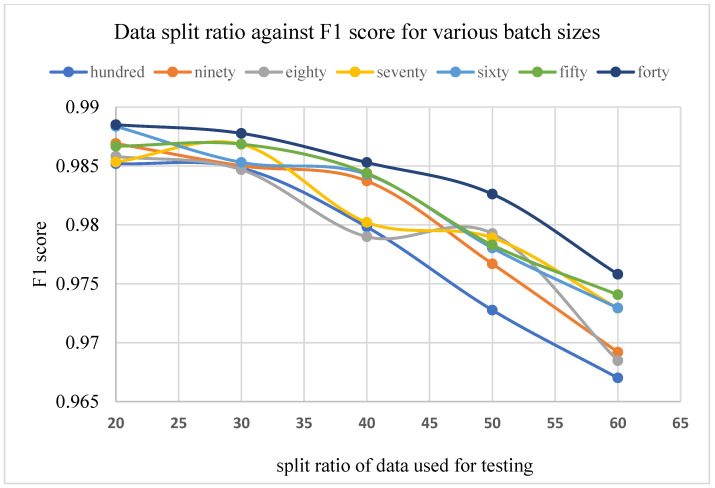
Split ratio against F1 score.

**Figure 7:**
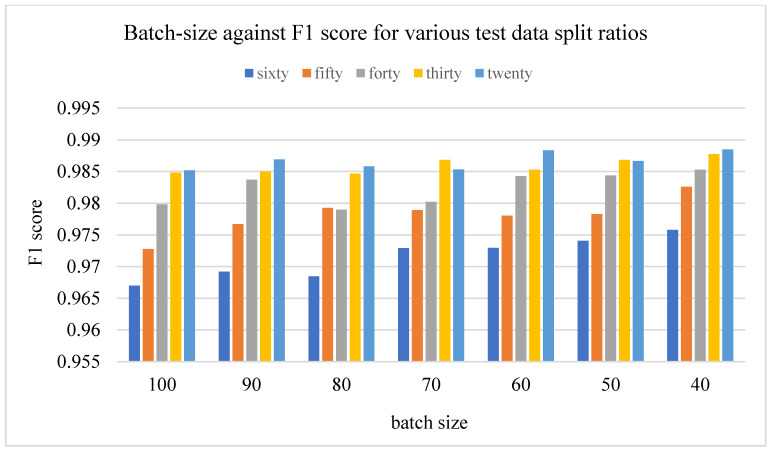
Batch size against F1 score for various validation split ratios.

The results of the F1 score for different train-validation data split ratios on the proposed residual neural network are displayed in Figure 6 and Figure 7 below.

The results in Figure 6 and Figure 7 show the relationship between the train-validation data split ratio and the F1 score of the networks. These results suggest that the split ratio had a great impact on the performance of the network. Figure 7 shows a clear distinction in the performance value as the train samples are increased.

For a batch size of 100 images, being the highest batch size value adopted for this research, train set values of 40, 50, 60, 70, and 80% of the entire dataset resulted in a performance of 0.9775, 0.9848, 0.9884, 0.9921, and 0.9937, respectively, out of a total value of 1, for a network depth of 152. The results on other network depths also show a similar pattern in performance with such data split ratios. This suggests that a split ratio of 80/20 is a good choice for plant leaf image recognition.

#### 3.2.3. Results on Different Batch Sizes

**Figure 8:**
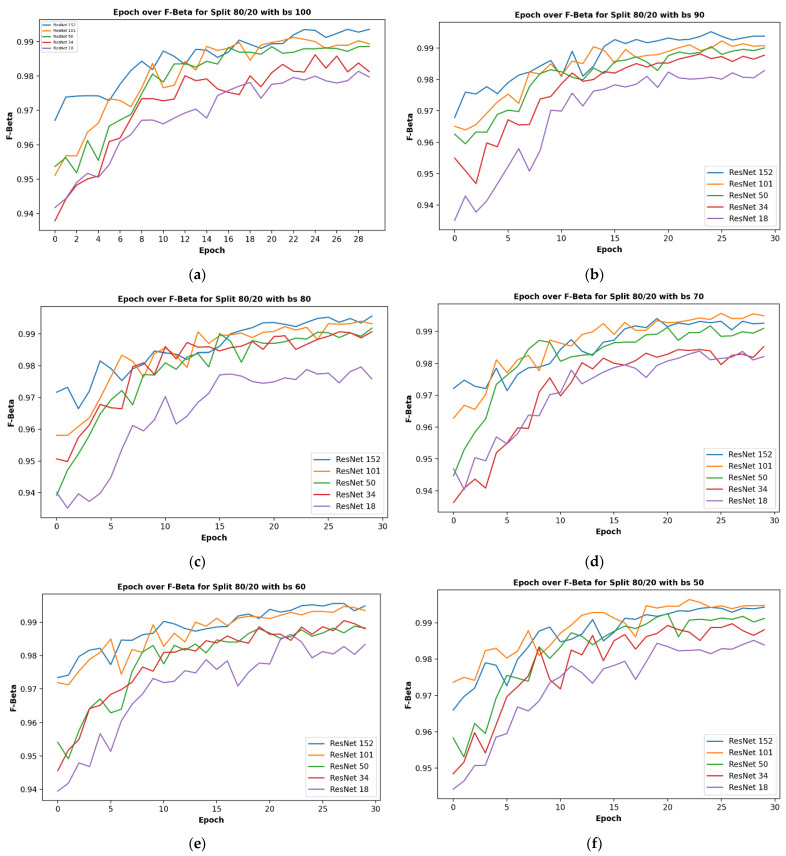
Showing the results on different train/test split ratios on various models used in our research: (**a**) showing epoch over F1 score for ResNet-18; (**b**) showing epoch over F1 score for ResNet-34; (**c**) showing epoch over F1 score for ResNet-50; (**d**) showing epoch over F1 score for ResNet-101; (**e**) showing epoch over F1 score for ResNet-152; (**f**) showing epoch over F1 score for ResNet-152 on a batch size of 80, this is where the best F1score of our research is located.

The effect of different batch sizes on the various models was studied and the results are described here. The results of the F1 score for different train-validation data split ratios on the proposed residual neural network are displayed in Figure 8 below. It displays the plot of the epoch over the F1 score for a batch size of 100, 90, 80, 70, 60, 60, and 50 images, respectively.

The results of the different batch sizes above do not show much difference in the overall performance of the F1 score at the end of the number of training epochs; however, the time taken was greatly influenced as is displayed in the next section.

#### 3.2.4. Results on Computing Time

**Figure 9:**
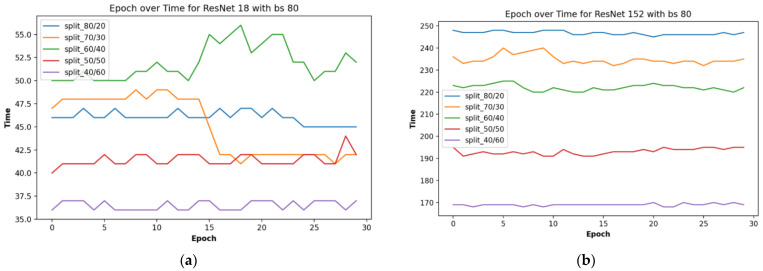
Epoch over time for depth of (**a**) 18, and (**b**) 152.

The results of time on the proposed residual neural network for plant leaf recognition are displayed in Figure 9, and Table 3 shows the time taken on various train-validation split ratios and batch sizes.

From Figure 9 above, the fastest of the networks was that of a depth of 18 layers, with a train-validation data split ratio of 40/60. Having more data for validation than for training made the network training and testing procedure faster, albeit our goal was not just for speed but also improved performance. Hence, the result of performance with the fastest time was not the optimized result we have. The split ratio of 80/20 was rather that with the highest performance in training and testing, as recorded in Table 2.

**Figure 10:**
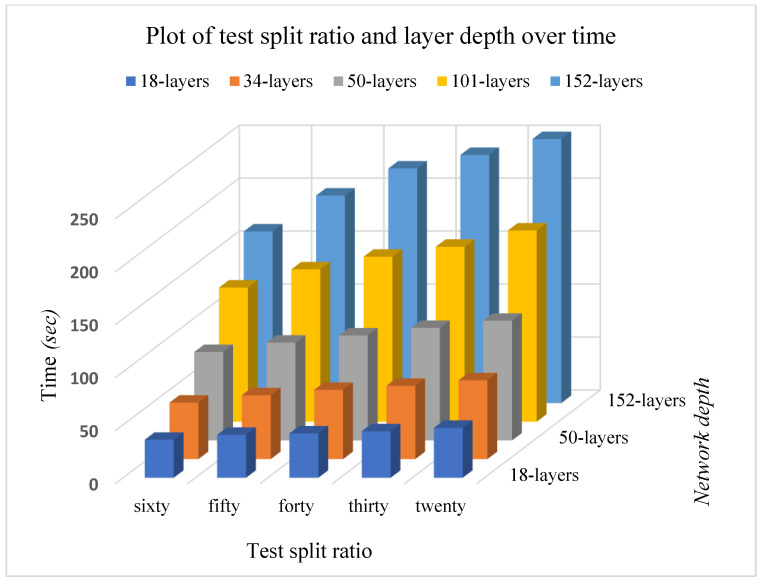
A plot of validation split ratio and layer depth over time.

Figure 10 shows the plot of the test split ratio and layer depth over time. This displays a more elaborate view of the relationship between time and other parameters such as split ratio and network depth.

**Figure 11:**
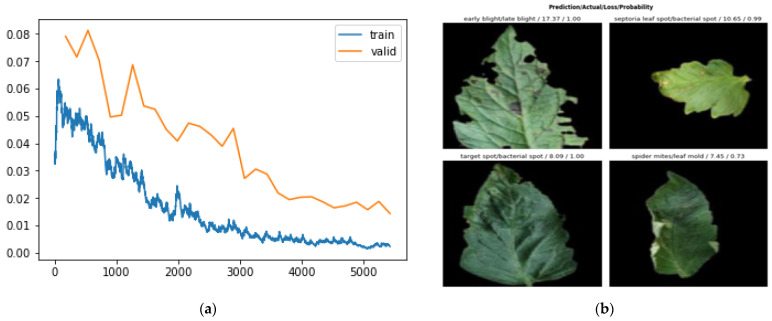
Showing the mistakes recorded by the model in our research: (**a**) showing the plot loss for ResNet-152; (**b**) showing the images the model predicted wrongly for ResNet-152, showing the predicted, ground truth, loss, and probability values.

The F1 score, being a more robust metric for recognition, combined both the recall and precision levels of the network. Here, we show how the network tried to maintain a considerably consistent F1 score for most of the training process with a +0.15 and −0.25 interval. The learning rate on this dataset had been seen to do well from 10^−5^ to 10^−3^. The best position to train a model has been estimated to fall along that axis. Afterward, as can be seen in the plot, as the learning rate increased, the model loss increased. Table 4 compares the performance of the proposed network on the PlantVillage dataset on various training and validation data split ratios. Figure 11 shows the areas of mistake recorded by the model in this research: (a) the plot loss for a network depth of 152; (b) the images the model predicted wrongly for a network depth of 152, detailing the predicted, ground truth, loss, and probability values.

The network, though having an outstanding performance, was not 100% perfect. From Figure 11 above, classes that were wrongly recognized are displayed. The first image shows how the model predicted a late blight leaf as an early blight class. Table 4 shows the result of the network on various train-validation split ratios. The early blight and late blight diseased leaves had a very striking resemblance and as such, most of the wrong predictions recorded by our method happened to fall in between the two leaf classes. Additionally, some of the spots that could be found on leaves were so close that they could constitute a challenge in perfect distinction, giving rise to an imperfect recognition, even to the human eye. However, more research on fine-grained distinct images is needed to get to that point.

**Figure 12:**
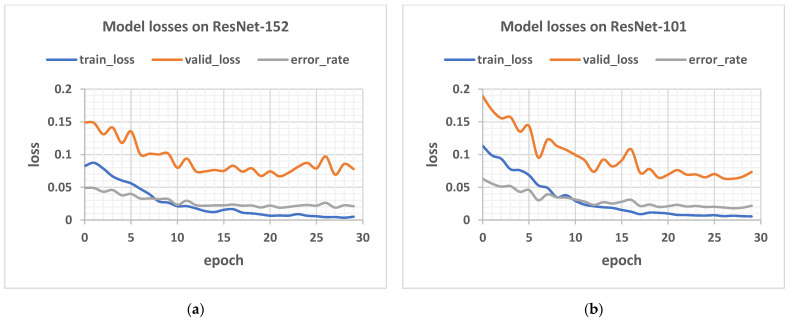

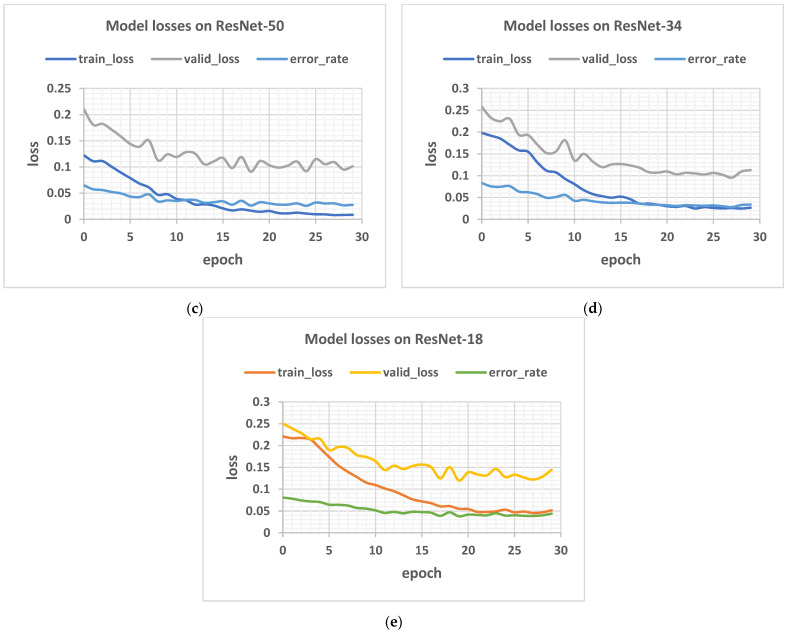
Showing the model losses and error rates for: (**a**) 152, (**b**) 101, (**c**) 50, (**d**) 34, and (**e**) 18 layers respectively.

Figure 12 below shows the plot of loss against epoch for the network of depth 152 layers on a train-validation split ratio of 40/60.

Table 5, Table 6, Table 7, Table 8 and Table 9 detail the performance of the proposed network on the PlantVillage dataset with various model depths of the residual neural network architecture on six different training and validation data split ratios. Table 5 compares the performance of our model on various model depths of the same architecture on a validation data split ratio of 60.

Table 6 compares the performance of our model on various model depths of the same architecture on a validation data split ratio of 50.

Table 7 compares the performance of our model on various model depths of the same architecture on a validation data split ratio of 40.

Table 8 compares the performance of our model on various model depths of the same architecture on a validation data split ratio of 30.

Table 9 compares the performance of our model on various model depths of the same architecture on a validation data split ratio of 20.

#### 3.2.5. Benchmark against Other Models

A summary of the related work carried out on the Flavia plant leaf image datasets and our result comparison is shown in Table 10.

**Figure 13:**
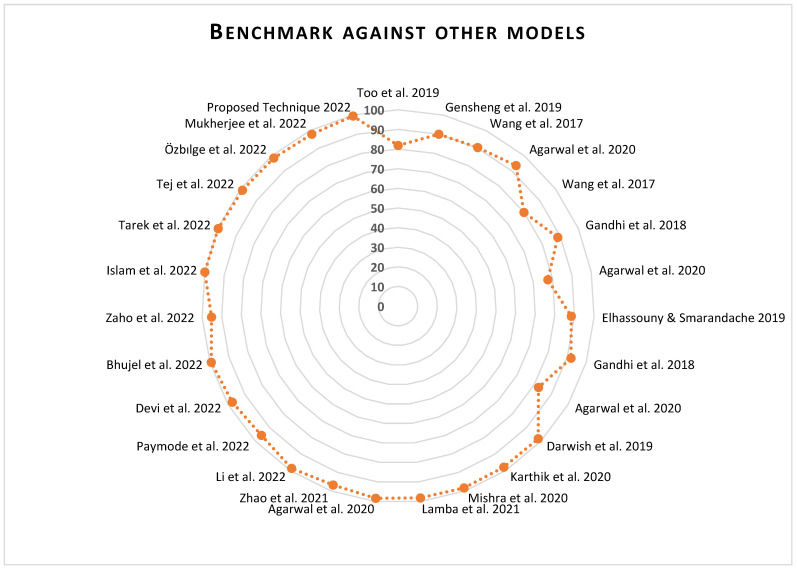
Benchmark against other models [27,47,48,49,58,59,60,61,62,63,64,65,66,67,68,69,70,71,72,73,74].

A summary of the related work on plant disease identification based on leaf images and the result comparison of some of them is shown in Table 11. As can be seen from both Table 11 and Figure 13, our model outperformed the previous models, surpassing that of Li et al. [58], which was the closest in performance, with a +0.75% performance gain. Whereby some authors used the accuracy metric to measure their performance, we recorded a much higher accuracy but chose to benchmark against our F1 score value, which is regarded as a much better form of performance measure for classification problems, since it combines both the precision and recall of the model in question.

Figure 13 below shows the chart of a comparison of our proposed technique and other techniques. A detailed summary of the technique, dataset and year of publication is just as displayed in Table 11 above. More discussion on the image is given in the Discussion section of this article.

## 4. Materials and Methods

### 4.1. Data Acquisition and Pre-Processing

#### 4.1.1. Datasets

1. The Flavia leaf dataset

**Figure 14:**
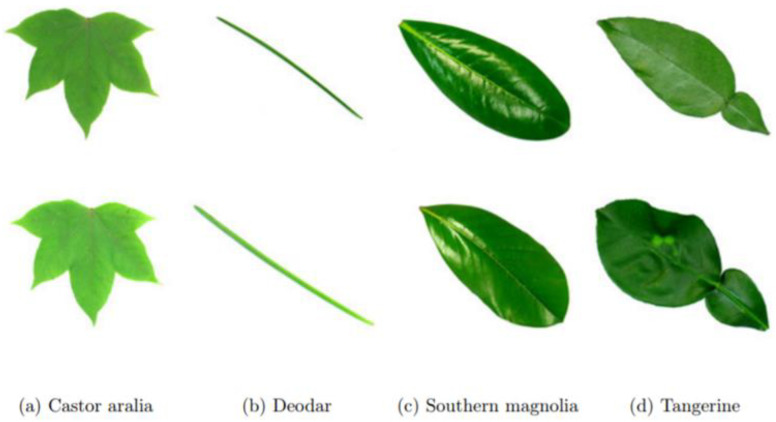
Samples from the Flavia leaf dataset.

The Flavia leaf dataset (download link: http://flavia.sourceforge.net/ (accessed on 13 November 2021)), introduced by Wu et al. [75], contains 1907 leaf images of size (1600 × 1200 pixels) obtained from 32 plant species on a white background, containing about 50–77 images per class. Figure 14 shows random sample images from the Flavia dataset used for this research work.

2. The tomato leaf dataset

**Figure 15:**
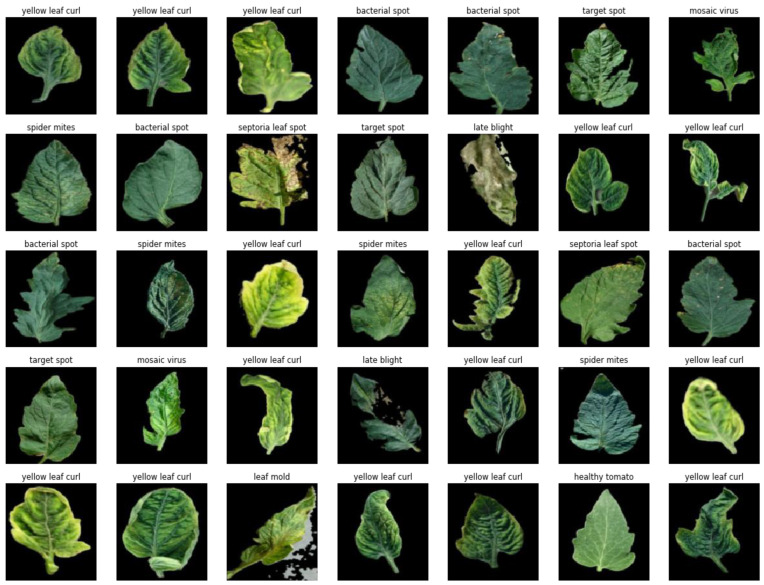
Samples from the tomato leaf dataset.

The tomato leaf dataset that was used in this research consists of images of diseased and healthy tomato plant leaves that were obtained from the publicly available PlantVillage [1] dataset, which is an open-access public resource for agriculture-related content. The entire dataset contains 54,306 images of plant leaves, which have a spread of 38 class labels assigned to them. However, the experiments in this research were narrowed down to only images of tomato plant leaves, which include nine types of tomato leaf diseases, and some healthy tomato leaves, making a total of 10 different categories for our research out of the entire 38 which are obtainable in the entire PlantVillage database. Our crop-specific data contained 18,160 images of tomato plant leaves, and each class is defined either with the corresponding name of the disease affecting the leaves in it or categorized as part of the healthy class. Figure 15 shows sample images from the PlantVillage dataset used for this research work.

From Figure 15, the various diseases can be categorized either as fungi, bacteria, mold, viruses, or mites. Four of the diseases, namely, early blight, late blight, leaf spot, and target spots are caused by fungi; the bacterial spot is caused by bacteria, the leaf mold is the cause of a mold disease, while both the tomato yellow leaf curl and the tomato mosaic are viral infections, and the spider mite is a mite disease. A brief description of each of these diseases is given below:

- Early blight is a fungal infection, and symptoms start as oval-shaped lesions with a yellow chlorotic region across the lesion; concentric leaf lesions may be seen on infected leaves.
- Late blight, being another fungal infection, affects all aerial parts of the tomato plant; initial symptoms of the disease appear as water-soaked green to black areas on leaves which rapidly change to brown lesions; fluffy white fungal growth may appear on infected areas and leaf undersides during wet weather.
- The leaf spot is another fungal infection. Infected plants exhibit bronzing or purpling of the upper sides of young leaves and develop necrotic spots; leaf spots may resemble those caused by bacterial spots, but a bacterial ooze test will be negative; leaves may cup downwards, and shoot tips may begin to die back.
- Septoria leaf spot is yet another fungal disease. Symptoms may occur at any stage of tomato development and begin as small, water-soaked spots or circular grayish-white spots on the underside of older leaves; spots have a grayish center and a dark margin, and they may coalesce.
- Leaf mold is still another fungal infection. The older leaves exhibit pale greenish to yellow spots (without distinguishable margins) on the upper surface, whereas, the lower portion of these spots exhibits green to brown velvety fungal growth. As the disease progresses, the spots may coalesce and appear brown. The infected leaves wither and die but stay attached to the plant.
- Bacterial spots are bacterial diseases, and lesions start as small water-soaked spots; lesions become more numerous and coalesce to form necrotic areas on the leaves giving them a blighted appearance; leaves drop from the plant, and severe defoliation can occur leaving the fruit susceptible to sunscald; mature spots have a greasy appearance and may appear transparent when held up to a light source; centers of lesions dry up and fall out of the leaf; blighted leaves often remain attached to the plant and give it a blighted appearance.
- Spider mites (two-spotted spider mites). Leaves stippled with yellow; leaves may appear bronzed; webbing covering leaves; mites may be visible as tiny moving dots on the webs or underside of leaves, best viewed using a hand lens; usually not spotted until there are visible symptoms on the plant; leaves turn yellow and may drop from the plant.
- Target spot is also caused by a fungus. The fungus infects all parts of the plant. Infected leaves show small, pinpoint, water-soaked spots initially. As the disease progresses, the spots enlarge to become necrotic lesions with conspicuous concentric circles, dark margins, and light brown centers. Whereas the fruits exhibit brown, slightly sunken flecks in the beginning, later the lesions develop a large, pitted appearance.
- Tomato mosaic virus is a viral infection. Symptoms can occur at any growth stage and any part of the plant can be affected; infected leaves generally exhibit a dark green mottling or mosaic; some strains of the virus can cause yellow mottling on the leaves; young leaves may be stunted or distorted; severely infected leaves may have raised green areas; dark necrotic streaks may appear on the petioles’ leaves.
- Tomato yellow leaf curl disease is another viral infection. The infected leaves become reduced in size, curl upward, appear crumpled, and show yellowing of veins and leaf margins.

#### 4.1.2. Data Pre-Processing

1. Data Augmentation 1—Image Transformations

Pre-processing steps are applied to cleanse and organize data before being fed into the model. By introducing a few distorted images into the training dataset, image transformations are used to increase the number of images in the dataset and reduce the chances of the model overfitting. The augmented images for the training data are created using standard image augmentation techniques such as flipping, Gamma correction, noise injection, PCA color augmentation, rotation, and scaling transformations. The images are each further resized to 128 before being fed to the training model.

2. Data Augmentation 2—CutMix

Let $x\;\in \;{\mathbb{R}}^{W\times H\times C}$ and y denote both the training image and the corresponding label of our tomato leaf image, respectively. The goal of the CutMix augmentator is to generate a new training sample $\left(\tilde{x},\tilde{y}\right)$ through the combination of two training samples, $\left(x_{A},y_{A}\right)$ and $\left(x_{B},y_{B}\right)$. The newly generated sample $\left(\tilde{x},\tilde{y}\right)$ is then used in training the model with its original loss function. 

We define the combining operation as

(5) $$\tilde{x}=M\;ʘ\;x_{A}+\left(1-M\right)\;ʘ\;x_{B}\;\tilde{y}=\lambda y_{A}+\left(1-\lambda \right)y_{B},\;$$

where **M** ∈ {0, 1}*^W×H^* denotes a binary mask indicating where to drop out and fill in from two images, **1** is a binary mask filled with ones and is an element-wise multiplication, and the combination ratio *λ* between two data points is sampled from the beta distribution *Beta*(*α*, *α*).

CutMix replaces an image region with a patch from another training image and generates locally natural images. CutMix is simple and incurs a negligible computational overhead as with existing data augmentation techniques; we can efficiently utilize it to train any network architecture. To sample the binary mask **M**, the bounding box coordinates $B=(r_{x},r_{y}$,$r_{w},r_{h})$ are first sampled indicating the cropping regions on $x_{A}$ and $x_{B}$. The region B in $x_{A}$ is removed and filled in with the patch cropped from B of $x_{B}$. The box coordinates are uniformly sampled according to:

(6) $$r_{x}\;∽\mathrm{Unif}\;\;\left(0,\;W\right),\;\;\;r_{w}=W\sqrt{1-\lambda },\;r_{y}\;∽\mathrm{Unif}\;\;\left(0,\;H\right),\;\;\;r_{h}=H\sqrt{1-\lambda },\;$$

making the cropped area ratio:

(7) $$\frac{r_{w}\;r_{h}}{W\;H}=1-\lambda \;$$

**Figure 16:**
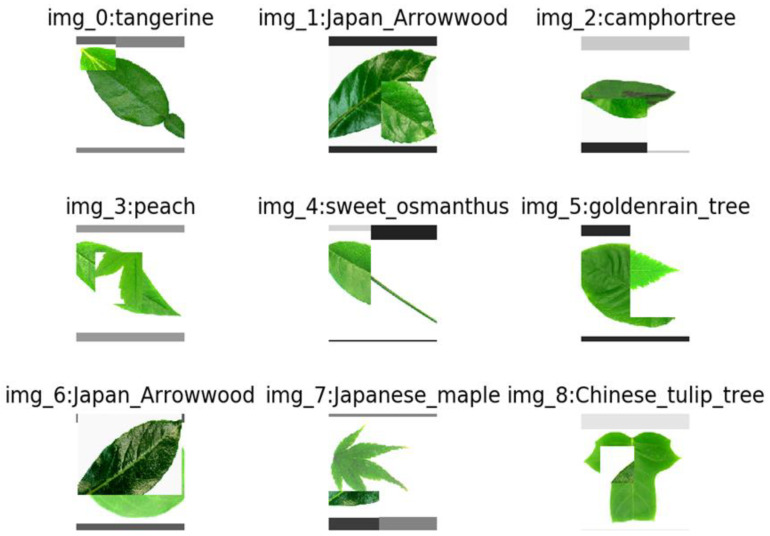
CutMix results from the Flavia dataset.

With the cropping region, the binary mask **M** ∈ {0, 1}*^W×H^* is decided by filling with 0 within the bounding box B, otherwise 1. In each training iteration, a CutMix-ed sample $\left(\tilde{x},\tilde{y}\right)$ is generated by combining two randomly selected training samples in a mini-batch according to Equation (5). Figure 16 shows a visualization of the CutMix operation on the Flavia dataset. The mixture of patch images on different class images can be seen.

### 4.2. Our Proposed Method

#### 4.2.1. Convolutional Neural Networks

A typical convolutional neural network architecture will contain some basic building blocks, some of which are referred to as layers. These make up the building block for our proposed network approach and are described in the following subsection.

1. Convolutional Layer

In this layer, convolutional operations are performed on the input to learn useful features. To this effect, a convolutional kernel slides along the input image with a certain stride and outputs convolution plus a bias generally known as a feature map. The input to this layer could be either an RGB image or the output feature of a preceding layer for a multilayer network. A convolutional kernel means that given an input image when it is processed, the weighted average of pixels in a small area of the input image becomes each corresponding pixel in the output image, and the weight is defined by a function, and hence, they share weights to reduce parameters in the network. This process can be expressed mathematically as:

(8) $$\begin{array}{c} X_{j}^{l}=f\left(\underset{i\in M_{j}}{\sum }Y_{i}^{l-1}K_{ij}^{l}+b_{j}^{l}\right) \end{array}$$

where $Y_{i}^{l-1}$ is the output of the *i-th* feature map in the l−1 layer, and $X_{j}^{l}$ is the input of the jth feature map in the l layer. $K_{ij}$ and $b_{j}^{l}$ are the convolutional kernel and bias in the l layer. f(.) is the activation function. Higher-level unique features can be identified through a series of increased convolution layers; hence, the need to go deeper.

2. Pooling Layer

After the convolution operation, there is a need to reduce the dimensions of the image for further processing. This process is known as down sampling or simply a pooling operation. This process can be expressed mathematically as:

(9) $$\begin{array}{c} X_{j}^{l}=f\left(\beta_{j}^{l}\cdot {\mathrm{down}}_{s}\left(X_{j}^{l-1}\right)+d_{j}^{l}\right) \end{array}$$

where $X_{j}^{l}$ represents the jth feature map in the l layer. $\beta_{j}^{l}$ and $d_{j}^{l}$ are the multiplicative factor and bias, respectively.$\cdot {\mathrm{down}}_{s}$ represents an under-sampling function. Under-sampling can be done in many forms, some of which are average pooling, maximal pooling (max pool), minimal pooling operation, and so on. In our work, we employed max pooling.

3. Fully Connected Layer

Each neuron in the fully connected layer is connected to all neurons in the feature map of the previous layer, and the output can be expressed as:

(10) $$\begin{array}{c} h_{W,b}\left(x\right)=f\left(W^{T}+b\right) \end{array}$$

where $h_{W,b}\left(x\right)$ is the output, and W represents the corresponding weights of the network. The inputs to the fully connected layer are mainly features extracted from the preceding layer. Each feature in the former layer represents different semantic information that is unique and important for the next layer.

#### 4.2.2. Transfer Learning Approach

The goal of transfer learning is to improve the target learners’ performance on target domains by transferring useful knowledge [76,77] from disparate but related source domains to the target at a lower computational cost. The reliance on a large number of target domain data for constructing target learners can thus be reduced. It has emerged as a popular and promising area of machine learning due to its wide range of application possibilities, especially in solving real-world problems [78,79,80,81] in a cheaper and more reliable method. The sphere of use of transfer learning is not few, coupled with its record of high results [82,83].

An approach in transfer learning is that the last few layers of the pre-trained network are replaced with new layers, such as a fully connected layer and a softmax classification layer, with the number of classes set to be equivalent to that of the new target dataset, which in our research is 10 for the number of tomato leaf classes. In our research, all the networks used were pre-trained on the imagenet dataset before they were re-trained to learn the features of the tomato leaf image dataset in order to correctly recognize them based on their different classes.

However, the challenge of maximizing its use with regard to modifying the learning rate to suit the target data still exists. It is discovered that one learning rate being used across all the layers of a model in transfer learning is not a good practice for transfer learning [84]. In each model, we unfreeze the layer and add a stack of one activation layer, one batch-normalization layer, and one dropout layer. All models are tested with the same dropout values, different learning rates, and varying batch sizes.

#### 4.2.3. Overall Architecture of the Proposed Method

**Figure 17:**
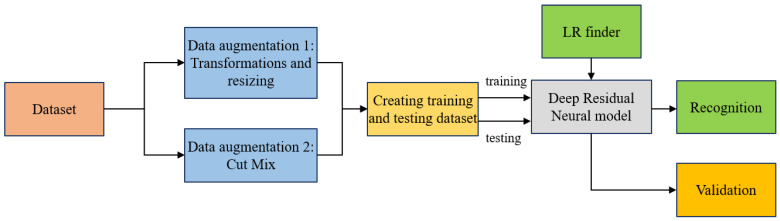
Workflow for the tomato leaf disease recognition system.

The proposed research network architecture consists of five networks designed similarly but with a different number of layers. Each layer consists of both an identity and a convolutional block. The identity block is the standard block used in ResNets and corresponds to the case where the input activation and output activation have the same dimensions. Figure 17 shows the entire workflow for the tomato leaf disease recognition system. 

**Figure 18:**
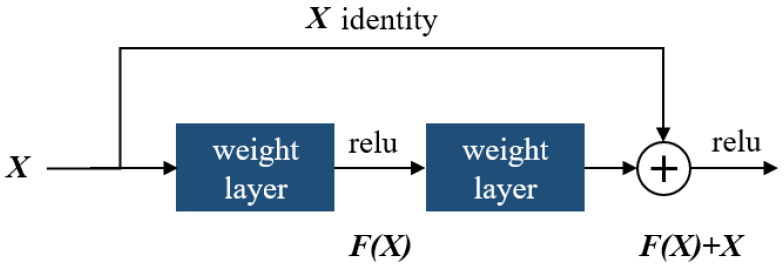
Showing the residual identity mapping for a residual neural network.

**Figure 19:**
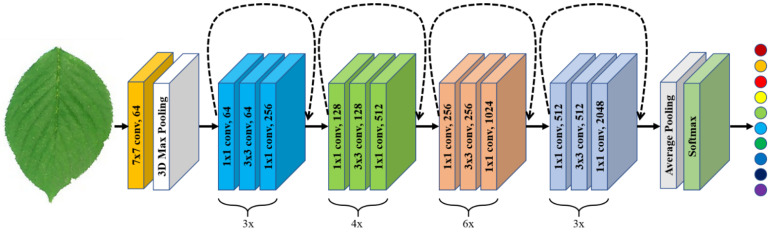
Simplified block diagram for the residual neural network.

The identity block is as shown in Figure 18, in which two hidden layers are skipped to avoid the vanishing gradient problem. The purpose of this block is to match input and output dimensions. The purpose of the identity block is to resize the input to a different dimension. Figure 18 shows the residual identity mapping for the residual neural network which is the basic building block for the network used in this research, while Figure 19 shows a simplified block diagram for the arrangement of the various blocks for the residual neural network [60] network used in this research.

Training deep learning architectures, whether it is a new model from scratch or via transfer learning is not without its difficulties. Learning algorithms based on artificial neural networks, and particularly deep learning for computer vision, may appear to include a plethora of bells and whistles, popularly known as hyperparameters [85]. To successfully train and debug them, more hyperparameters should be adjustable, and this has made it possible for one to obtain more interesting results. Making sure we have the right learning rate is one of the most important things we can do when training a model. If our learning rate is too low, training our model may take many, many epochs. For this research, all our five networks were pre-trained with the ImageNet dataset and then fine-tuned in the manner described below for re-training on our tomato leaf images.

We implement discriminative learning rates [86] in the process of training our model on plant leaf images. Discriminative fine-tuning is a fine-tuning strategy introduced by universal language model fine-tuning (ULMFiT) [86] for the implementation of natural language processing (NLP). Instead of the same learning rate being used across all the layers of our model, this allows us to tune each layer with a different learning rate that is efficient and suits it better than any other learning rate. Since the deepest layers of pre-trained models may not require so high a learning rate as the final ones will require, we will use different learning rates for them as described by [84,87].

To preserve the quality of those pre-trained weights even after unfreezing them, some of the added parameters are tuned for a few epochs, and naturally, we would not expect the best learning rate for those pre-trained parameters to be as high as the best learning rate for the randomly added parameters. Keep in mind that the pre-trained weights were trained on millions of images over hundreds of epochs, so they have learned rich features from being previously trained on large datasets. Our experiment was carried out with a few specific detailed goals in mind, they are:

1. To research the role of depth on the performance of such models, various residual convolutional neural networks, having different layers, such as ResNet-18, ResNet-34, ResNet-50, ResNet-102, and ResNet-152 were tested.
2. Various train/test data split ratios were experimented on to determine the optimum value of the train/test split ratio for such a research area.
3. Different batch sizes were selected based on the capacity of the GPU system obtainable in the laboratory to test for the influence of batch size on the training and if so on the result of the training and test processes of the model.
4. The discriminative learning process was studied to determine the best learning rates to select in re-training the various models to achieve an optimal training process from one domain of data to a new domain of datasets: specific to this research is the tomato plant leaf dataset for disease recognition.

### 4.3. Training Procedure

#### 4.3.1. Tuning the Learning Rate Schedule

**Figure 20:**
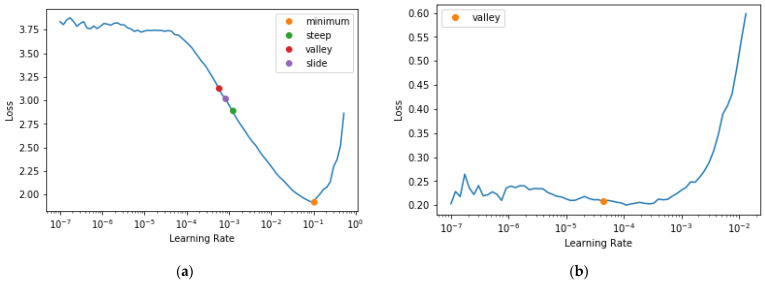
Showing results of the learning rate finder on the network: (**a**) shows the learning rate find result before unfreezing for the network of depth 152; (**b**) shows the result of the second learning rate find after unfreezing the model and training briefly on the first selected learning rate.

We began with an extremely low learning rate, and used it for one mini-batch, then determined the losses and increased the learning rate by a certain percentage, i.e., doubling it each time. Then, we ran another mini-batch and repeated the procedure above until the loss worsened rather than improved. This is the point where we realized we had gone too far. Then, we chose a learning rate that was slightly lower than this point. We used discriminative learning rates and a gradual unfreezing of the network to re-train the network using the tomato leaf images. The learning rate tool in fast.ai (fastai is a deep learning library which provides practitioners with high-level components [88]) was employed to return a plot of learning rate versus loss (cross-entropy) and to identify the optimal learning rates to use with the Adam optimizer. Figure 20 shows the plot of the learning rate curve as we trained the network. We used smaller learning rates in the early layers to permit the weights in those layers to change in a slower pattern than those from the later layers: 1 × 10^−4^, 1 × 10^−3^, and 1 × 10^−2^ for the first, middle, and last layers, respectively. While training, for the first three epochs, only the final layers were trained, while all prior layers were frozen. Subsequently, all layers were unfrozen and trained for an additional 27 epochs.

We can see from plot Figure 20a that in the range of 1 × 10^−6^ to 1 × 10^−4^, nothing happened and the model did not train. Then, the loss started to decrease until it reached a minimum at 1 × 10^−1^, and then it increased again. This showed that a learning rate greater than 1 × 10^−1^ was high and will make training diverge, but a learning rate of 1 × 10^−1^ was already too high for training; hence, we needed to select a better rate. Figure 20 shows us four different points from our learning rate finder: valley, slide, steep, and minimum points, respectively. With such, we can select an appropriate value for the learning rate. However, that is not the end of the training procedure. Our goal is to train the whole network without breaking the pre-trained weights.

**Figure 21:**
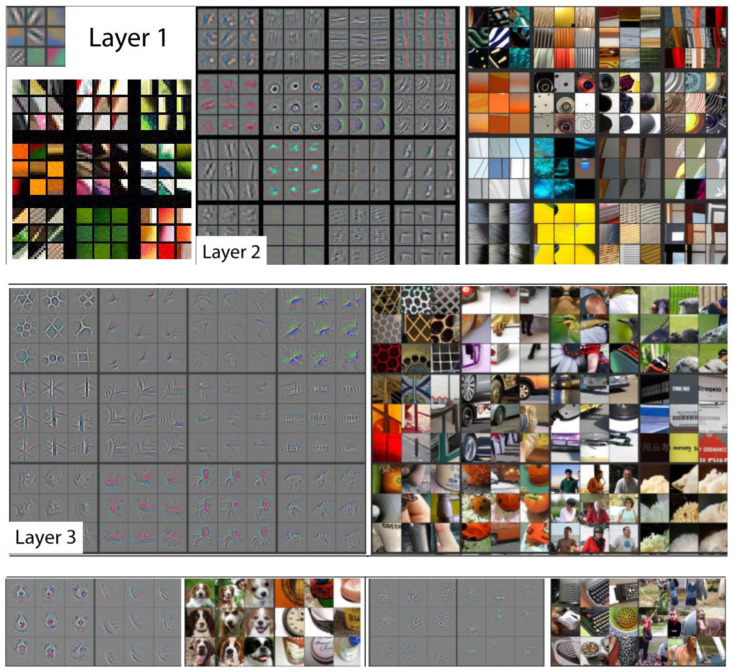

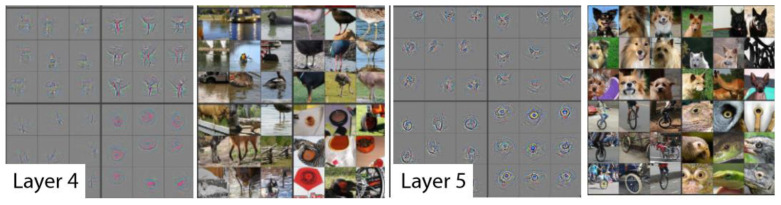
Activations of a convolutional neural network by layers.

Figure 21 shows activations of a convolutional neural network by layers, visually demonstrating what is learned by the different layers of a model [89].

Because different layers in a neural network capture different types of information, there is a need for them to be fine-tuned to different extents. Instead of using the same learning rate for all layers of the model, the practice of discriminative fine-tuning allows for the tuning of each layer in a neural network with different learning rates. For a proper context, the regular SGD update of a model’s parameters *θ* at time step *t* looks can be represented by the following:

(11) $$\begin{array}{c} \theta t=\theta t-1-\eta \;\cdot \;\nabla \theta J\left(\theta \right) \end{array}\;$$

where *η* is the learning rate and ∇*θJ*(*θ*) represents the gradient with regard to the objective function of the model in question.

For discriminative fine-tuning, we split the parameters *θ* into {*θ*^1^,...,*θ^L^*}, where *θ^l^* contains the parameters of the model at the *l*-th layer and *L* is the number of layers in the entire model.

Similarly, we obtain {*η*^1^,...,*η^L^*}, where *η^l^* signifies the learning rate of the *l*-th layer.

The SGD update with discriminative fine-tuning can then be represented by the following:

(12) $$\begin{array}{c} \theta tl=\theta tl-1-\eta l\;\cdot \;\nabla \theta lJ\left(\theta \right) \end{array}\;$$

Empirically, it has been found to be the best practice to first of all choose the learning rate *η^L^* of the last layer by fine-tuning only the last layer and using *η^l^*^−1^ = *η^l^*/2.6 as the learning rate for lower layers.

#### 4.3.2. Unfreezing and Re-Tuning the Learning Rate Schedule

**Figure 22:**
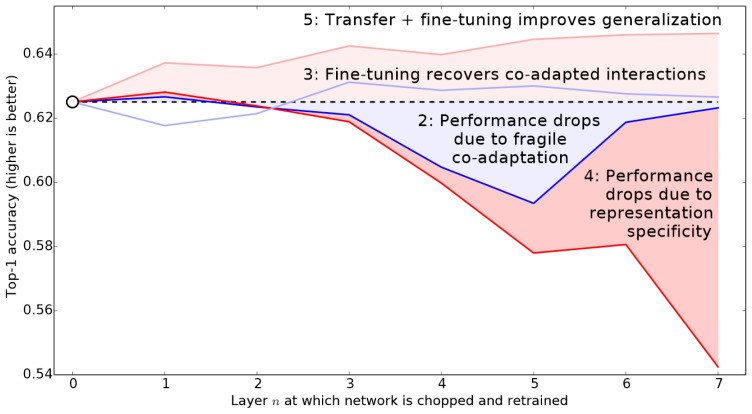
Layer depth against performance in training methods on transfer learning.

As we went about fine-tuning the model, the deepest layers of our pre-trained ResNet model did not need as high a learning rate as the last ones, so we should probably use different learning rates for those—this is known as using discriminative learning rates. It is based on the idea that we use a lower learning rate for the early layers of the neural network, and a higher learning rate for the later layers, especially the randomly added layers that suit the problem to be solved. It derives its foundation from the insights drawn from the proposal of Jason Yosinski et al. [87] as summarized in Figure 22 below, which shows the impact different layers make and training methods on the transfer learning approach in applications of deep learning models.

The learning rate finder selects a minibatch of images, determines the gradient for the minibatch, and then steps the weights based on the learning rate and the gradient. Leslie Smith initially set a very low learning rate for the first minibatch before gradually raising it. We chose the steep or minimal point divided by a value of 10 because the minimum was where learning stops, but the steepest point was where learning was most effective. The early layers did not need to be modified at all; however, the later layers did need to be adjusted to fit the tomato leaf images. Therefore, we used discriminative learning rates and set a smaller learning rate for the early layers and a greater learning rate for the latter layers.

### 4.4. Experimental Setup

The training and testing processes in this experiment were implemented on Pytorch using fastai library [88]; the scikit-learn, pillow, and OpenCV libraries were used, all of which are written in Python. The model was trained and tested on an NVIDIA DGX-1 V100 with 8X Tesla V100 GPUs with a performance of one petaFLOP.

To carry out this research, a computer system was used to carry out the analysis processes. The computer system has the following specifications: Windows 10, 64-bit, Intel Core i7-4720 CPU @ 2.60 GHz, RAM 32 GB and GPU Nvidia GeForce GTX 1050 4 GB dedicated memory, and Python 3.7 on Anaconda. The PC was used on a Linux-based DELL PowerEdge T640 Tower Server with CUDA-based video cards GTX 1080TI, each GPU Video memory was 11Gb, with a storage memory of 10TB Hard Drive and 3320 GB SSD. Table 12 and Table 13 detail the setup of the system used and the parameters used in running the model.

## 5. Discussion

Using data from ten different classes, the effect of different depths, and batch sizes (depth = 18, 34, 50, 101, and 152 and BS = 40, 50, 60, 70, 80, 90, and 100) and parameter values on their performance in adequately recognizing healthy tomatoes and other diseased classes were investigated in this study. The research was carried out using transfer learning and a pre-trained residual neural network. They performed well on the training and test data, according to the results. However, the steady state in the test data was observed to be delayed as the batch size value increased. The highest recognition accuracy value was 99.51% for depth = 152, with a train/validation data split size of 80/20 and a batch size of 40. According to the findings, the batch size value has no significant effect on overall performance but increasing the batch size value delays obtaining stable results.

Table 11 shows a detailed comparison of our proposed technique with other state-of-the-art models. As can be seen from Table 11 and Figure 13, our model outperformed the previous models, surpassing that of Li et al. [58], which was the closest in performance, with a +0.75% performance gain, and achieved a favorable performance with a +3.79% improvement from that of Paymode et al. [69] and other previous models. However, most recent models, such as Islam et al. [84] and Tarek et al. [71] outperformed our method, recording 100% and 99.81% accuracy. Whereby some authors used the accuracy metric to measure their performance, we recorded a much higher accuracy but chose to benchmark against our F1 score value, which is regarded as a much better form of performance measure for classification and recognition problems, since it combines both the precision and recall of the model in question. 

## 6. Conclusions

Discriminative learning was implemented in training our proposed networks, having diverse layers, such as 18, 34, 50, 102, and 152 layers, respectively. The results show that the network with the highest depth produced the best performance, suggesting that deeper networks could still be better in deep learning. Five train/test data split ratios of 40/60, 50/50, 60/40, 70/30, and 80/20 were experimented on to determine the optimum value of the train/test split ratio for such a research area. The split ratio of 80/20 resulted in the optimal solution, with 70/30 coming after it. Different batch sizes were selected based on the capacity of the GPU system obtainable in the laboratory to test for the influence of batch size on the training, and if so on the result of the training and test processes of the model. The charts show a slight increase in performance for lesser batch sizes, while the most notable effect of the batch sizes was in speeding up the training process. The discriminative learning process was studied to determine the best learning rates to select in re-training the network during transfer learning, and the results show how effective it is to pre-train, determine a range of suitable learning rates, and re-train the entire network based on the discriminative learning rates to achieve a faster and more efficient performance. The data augmentation also proved to be effective, as the results were a little bit improved with the augmentation process.

## Figures and Tables

**Table 1:** List of tomato plant pathogens present in the Mediterranean basin.

Pathogen Group	Pathogen Name	Reference
Fungi	*Alternaria solani*, *Botrytis cinerea*, *Cladosporium fulvum*, *Colletotrichum coccodes*, *Fusarium oxysporum*, *Fusarium clavum*, *Leveillula taurica*, *Oidium lycopersici*, *Pseudoidium neolycopersici*, *Pyrenochaeta lycopersici*, *Rhizoctonia solani*, *Septoria lycopersici*, *Sclerotinia sclerotiorum*, *Sclerotium rolfsii*, *Stemphylium* spp., *Verticillium dahliae*	[4,6]
Oomycetes	*Phytophthora infestans*, *Phytophthora nicotianae*, *Phytophtora cryptogea*, *Pythium debaryanum*, *Pythium sylvaticum*	[7]
Bacteria	*Clavibacter michiganensis* subsp. *michiganensis*, *Erwinia carotovora* subsp. *carotovora*, *Pseudomonas corrugata*, *Pseudomonas mediterranea*, *Pseudomonas syringae pv. tomato*, *Ralstonia solanacearum*, *Xanthomonas axonopodis pv. vesicatoria*	[7]
Phytoplasma	*Candidatus* Phytoplasma solani	[8]
Viruses	Alfalfa mosaic virus, Chickpea chlorotic dwarf virus, Cucumber mosaic virus, Eggplant mottled dwarf virus, Parietaria mottle virus, Pelargonium zonate spot virus, Pepino mosaic virus, Potato virus Y, Southern tomato virus, Tobacco mosaic virus, Tomato brown rugose fruit virus, Tomato chlorosis virus, Tomato infectious chlorosis virus, Tomato leaf curl New Delhi virus, Tomato mosaic virus, Tomato spotted wilt virus, Tomato torrado virus, Tomato yellow leaf curl virus, Tomato yellow leaf curl Sardinia virus	[4,9]
Viroids	Potato spindle tuber viroid, Tomato apical stunt viroid	[8]

**Table 2:** Performance on various split ratios.

Batch Size	Performance (%)				
	40/60	50/50	60/40	70/30	80/20
100	0.977447	0.984816	0.988378	0.992139	0.993655
90	0.97957	0.987927	0.993078	0.99241	0.99521
80	0.981349	0.987848	0.990103	0.992641	0.995611
70	0.984734	0.986958	0.988716	0.994496	0.994071
60	0.984338	0.987595	0.994316	0.993444	0.995579
50	0.987115	0.9893	0.993566	0.994769	0.994317
40	0.985295	0.990085	0.993045	0.994083	0.995048

**Table 3:** Time taken.

Batch Size	Time (s)				
	40/60	50/50	60/40	70/30	80/20
100	162	196	221	234	249
90	179	188	218	237	253
80	169	193	222	235	247
70	176	188	211	232	240
60	172	193	217	236	259
50	188	208	221	242	275
40	200	204	226	248	281

**Table 4:** Results for various train-validation split ratios.

Train Split (%)	Validation Split (%)	Train Loss	Valid Loss	Accuracy	Recall	Precision	F1 Score
90	10	0.052291	0.07908	0.976046	0.972897	0.972338	0.97166
80	20	0.049548	0.071533	0.976597	0.973958	0.973845	0.97321
70	30	0.045366	0.081245	0.97109	0.967464	0.966935	0.966463
60	40	0.042245	0.070298	0.975771	0.972307	0.97289	0.971938
50	50	0.033666	0.049622	0.984857	0.981165	0.982264	0.981518
40	60	0.002324	0.014366	0.996421	0.995781	0.995451	0.995611

**Table 5:** Network depth on validation split = 60.

Network Depth	Error Rate	F1 Score	Time (s)
18	0.037812	0.953628	35
34	0.028084	0.964552	53
50	0.025881	0.96916	83
101	0.01808	0.977994	126
152	0.018906	0.977447	160

**Table 6:** Network depth on validation split = 50.

Network Depth	Error Rate	F1 Score	Time (s)
18	0.027423	0.967306	40
34	0.024559	0.968959	59
50	0.018612	0.978017	91
101	0.015419	0.981207	139
152	0.013216	0.984816	187

**Table 7:** Network depth on validation split = 40.

Network Depth	Error Rate	F1 Score	Time (s)
18	0.021338	0.974903	41
34	0.015694	0.979904	63
50	0.015556	0.98172	97
101	0.010187	0.988444	152
152	0.009637	0.988378	217

**Table 8:** Network depth on validation split = 30.

Network Depth	Error Rate	F1 Score	Time (s)
18	0.016153	0.978283	43
34	0.011013	0.985728	69
50	0.011197	0.986037	105
101	0.009728	0.987897	160
152	0.006608	0.992139	228

**Table 9:** Network depth on validation split = 20.

Network Depth	Error Rate	F1 Score	Time (s)
18	0.015419	0.981407	47
34	0.011564	0.986212	73
50	0.009086	0.988667	112
101	0.006883	0.991295	179
152	0.005507	0.993655	243

**Table 10:** Comparison of the proposed technique with the existing prevalent approaches on the Flavia dataset.

Technique	Year	Objective	# Images	Methods	Accuracy (%)
Keivani et al. [50]	2020	Flavia dataset	1907	Decision Tree	98.58
Li et al. [51]	2021	Flavia dataset	1907	DenseNet201	98.69
Kanda et al. [52]	2021	Flavia dataset	1907	DL + Logistic Regression	99.0
Thanikkal et al. [53]	2022	Flavia dataset	1907	DL	99.0
Twum et al. [54]	2022	Flavia dataset	1907	Log Gabor Filters	97.0
Gajjar et al. [55]	2022	Flavia dataset	1907	Extreme learning machines	99.10
Goyal et al. [56]	2022	Flavia dataset	1907	Hierarchical cluster	96.24
Ganguly et al. [57]	2022	Flavia dataset	1907	ResNet + Bonferroni mean operator	98.7
Proposed Network	2022	Flavia dataset	1907	ResNet + Discriminative Learning	99.23

**Table 11:** Comparison of the proposed technique with the existing prevalent approaches on the tomato leaf dataset.

Technique	Year	Objective	# Images	Methods	Accuracy (%)
Too et al. [59]	2019	Plant leaf disease	54,306	VGG16	81.83
Gensheng et al. [60]	2019	Tea leaf disease	4980	VGG16	90
Wang et al. [61]	2017	Plant leaf disease	54,306	VGG16	90.4
Agarwal et al. [62]	2020	Tomato leaf disease	18,160	VGG16	93.5
Wang et al. [61]	2017	Plant leaf disease	54,306	Inception-V3	80
Gandhi et al. [63]	2018	Plant leaf disease	56,000	Inception-V3	88.6
Agarwal et al. [62]	2020	Tomato leaf disease	18,160	Inception-V3	77.5
Elhassouny & Smarandache [64]	2019	Tomato leaf disease	7176	MobileNet	88.4
Gandhi et al. [63]	2018	Plant leaf disease	56,000	Mobilenet	92
Agarwal et al. [62]	2020	Tomato leaf disease	18,160	Mobilenet	82.6
Darwish et al. [65]	2019	Maize leaf disease	15,408	VGG19	98.2
Karthik et al. [27]	2020	Tomato leaf disease	5452 (4 classes)	ResNet + DenseNet	98
Mishra et al. [66]	2020	Corn leaf disease	3703	CNN	98.4
Lamba et al. [67]	2021	Tomato leaf disease	16,012	CNN	98.2
Agarwal et al. [62]	2020	Tomato leaf disease	18,160	CNN	98.4
Zhao et al. [68]	2021	Tomato leaf disease	18,160 (10 classes)	ResNet50 + SeNet	96.81
Li et al. [58]	2022	Tomato leaf disease	4240	FWDGAN + B-ARNet	98.75
Paymode et al. [69]	2022	Tomato leaf disease		VGG16	95.71
Devi et al. [47]	2022	Tomato leaf disease	9281	DensNet + Attention mechanism	97.56
Bhujel et al. [48]	2022	Tomato leaf disease	19,510	Lightweight Attention-Based CNN	99.34
Zaho et al. [49]	2022	Tomato leaf disease	18,160	Spatial attention with CNN	95.20
Islam et al. [70]	2022	Tomato leaf disease	15,989	cGAN + CNN + Logistic Regression	100
Tarek et al. [71]	2022	Tomato leaf disease	16,004	MobileNetV3	99.81
Tej et al. [72]	2022	Pepper and Tomato leaf diseases	488	CNN	98.85
Özbılge et al. [73]	2022	Tomato leaf disease	18,160	Compact CNN	98.49
Mukherjee et al. [74]	2022	Tomato leaf disease	10,839 (7 classes)	Gray Wolf + MobileNetV2	98
Proposed Technique	2022	Tomato leaf disease	18,160	ResNet + Discriminative Learning	99.51

**Table 12:** Configuration of the machine used.

Name	Parameter
Memory	32 GB
Processor	Intel(R) Xeon(R) Silver 4114 CPU @ 2.20 GHz
Server model	DELL PowerEdge T640 Tower Server
Graphics	CUDA-based video cards 4X 1080TI; GPU Video memory of 11 Gb
OS	Linux
Language	Python 3
Framework	Pytorch

**Table 13:** Configuration of the machine used.

Name	Parameter
Solver type	Adam
Batch sizes	20, 30, 40, 50, 60, 70, 80, 90, 100
Image input size	256 × 256
Train/Test-split ratio	40/60, 50/50, 60/40, 70/30, 80/20
Learning rate	Discriminative ranges
Drop out	0.5

## Data Availability

The PlantVillage dataset is publicly available online at https://github.com/spMohanty/PlantVillage-Dataset (accessed on 13 November 2021).
